# The Accuracy of Sequence-Specific Oligonucleotide and Real-Time Polymerase Chain Reaction HLA Typing in Determining the Presence of Pre-Transplant Donor-Specific Anti-HLA Antibodies and Total Eplet Mismatches for Deceased Donor Kidney Transplantation

**DOI:** 10.3389/fimmu.2022.844438

**Published:** 2022-06-20

**Authors:** Nicholas G. Larkins, Lloyd D’Orsogna, Anne Taverniti, Ankit Sharma, Aron Chakera, Doris Chan, Anoushka Krishnan, Germaine Wong, Wai H. Lim

**Affiliations:** ^1^ Department of Nephrology, Perth Children’s Hospital, Perth, WA, Australia; ^2^ School of Medicine, University of Western Australia, Perth, WA, Australia; ^3^ Department of Clinical Immunology, Fiona Stanley Hospital, Perth, WA, Australia; ^4^ Centre for Kidney Research, The Children’s Hospital at Westmead, Sydney, NSW, Australia; ^5^ Sydney School of Public Health, University of Sydney, Sydney, NSW, Australia; ^6^ Department of Renal Medicine and National Pancreas Transplant Unit, Westmead Hospital, Sydney, NSW, Australia; ^7^ Department of Renal Medicine, Sir Charles Gardiner Hospital, Perth, WA, Australia; ^8^ Department of Renal Medicine, Royal Perth Hospital, Perth, WA, Australia

**Keywords:** epitopes, histocompatibility, histocompatibility typing, genotyping techniques, kidney transplantation, organ transplantation

## Abstract

High resolution human leukocyte antigen (HLA) typing is important in establishing eplet compatibility and the specificity of donor-specific anti-HLA antibodies (DSA). In deceased donor kidney transplantation, high resolution donor HLA typing may not be immediately available, leading to inaccuracies during the organ allocation process. We aimed to determine the concordance and agreement of HLA-Class I and II eplet mismatches calculated using population frequency based allelic haplotype association (linkage disequilibrium, LD) from sequence-specific oligonucleotide (SSO) and real-time polymerase chain reaction (rtPCR) donor HLA typing (available at time of donor kidney allocation) compared to high-resolution Next Generation Sequencing (NGS) donor typing. NGS high resolution HLA typing were available for all recipients prior to donor kidney allocation. A cohort of 94 deceased donor-recipient pairs from a single Western Australian center were included (77 individual donors typed, 55 local and 22 interstate). The number of class I (HLA-A+B+C) and class II (HLA-DRB1+DRB3/4/5+DQB1+DQA1+DPB1+DPA1) eplet mismatches were calculated using HLAMatchmaker, comparing LD- and NGS-HLA typing. The accuracy in assigning pre-transplant DSA was compared between methods. The concordance correlation coefficient (95%CI) for HLA-class I and II eplet mismatches were 0.994 (0.992 to 0.996) and 0.991 (0.986 to 0.993), respectively. The 95% limits of agreement for class I were -1.3 (-1.6 to -1.1) to 1.4 (1.2 to 1.7) and -4.8 (-5.7 to -3.9) to 5.0 (4.1 to 5.9) for Class II. Disagreement between the two methods were present for 11 and 37 of the Class I and II donor/recipient pairs. Of which, 5 had a difference of ≥5 class II eplet mismatches. There were 34 (36%) recipients with potential pre-transplant DSA, of which 8 (24% of recipients with DSA) had indeterminate and ultimately false positive DSA assigned by donor LD-typing. While the concordance between NGS- and LD-typing was high, the limits of agreement suggest meaningful differences between these two techniques. The inaccurate assignment of DSA from donor LD-typing may result in associated HLA being considered unacceptable mismatches, inappropriately precluding candidates’ access to transplantation. Accurate imputation of two-field HLA alleles based on LD from SSO and rtPCR HLA typing remains a substantial challenge in clinical practice in-lieu of widely available, rapid, high-resolution methods.

## Introduction

Human leukocyte antigen (HLA) compatibility between donors and recipients remains the cornerstone of immunological risk assessment in kidney transplantation, with incremental mismatches at the HLA class I and II alleles resulting in an increased risk of allograft failure and development of donor-specific anti-HLA antibodies (DSA) (1). The evolution of HLA typing from serological-based techniques to polymerase chain reaction (PCR)-based methods has substantially improved the characterization and understanding of the HLA system, including allele-level resolution data which better defines HLA compatibility and specificity of DSA (2). These high-resolution data can then be used to estimate conformational similarities between HLA molecules based on predicted exposed amino acid sequences eliciting an immune response, known as eplets (3).

The most common HLA typing methods used in clinical practice are molecular methods, such as by sequence-specific oligonucleotide (SSO) and real time PCR (rtPCR) sequence specific primers, and Next Generation Sequencing (NGS). Molecular methods have the advantage of a shorter turn-around time and remain the standard practice used in deceased donor organ allocation in Australia, the United States and Europe, providing low-intermediate resolution typing of 1 or 2 fields for a few, but not all, relevant HLA loci. NGS produces high resolution HLA typing without allelic ambiguity at all relevant HLA loci, and up to 4 field-typing for some alleles. High-resolution HLA typing is the basis upon which eplet matching was developed. When low-intermediate resolution HLA typing has been used, only 1 to 2 field typing is available and only at some alleles, so the eplet prediction software uses a process of imputation based on linkage disequilibrium to determine the most likely high-resolution alleles equivalent. This introduces uncertainty into the process and error in determining eplet compatibility for populations for which there is limited high-resolution HLA population frequency data, namely minority groups who tend to be already disadvantaged by the structure and function of organ allocation programs. In addition, the availability of low-intermediate resolution HLA typing at the time of organ allocation can lead to uncertainty about the specificity of potential DSAs, resulting in potentially appropriate donors being declined.

In this study we aimed to determine the concordance and agreement of the HLA-Class I and II eplet mismatches calculated using population frequency based allelic haplotype association from high resolution HLA typing for donor/kidney transplant recipient pairs. We also sought to examine the accuracy of using low-intermediate resolution HLA donor typing at the time of donor kidney allocation to identify actual pre-existing DSA in transplant recipients.

## Materials and Methods

### Study Cohort

Of 145 deceased donor kidney transplants undertaken at Sir Charles Gairdner Hospital, Perth, Western Australia, between 2017 and 2019, 94 (65%) recipients and corresponding donors had high resolution HLA typing at the HLA-A, -B, -C, -DRB1, -DRB3/4/5, -DQA1, -DQB1, -DPA1 and -DPB1 alleles and were included in this study. While all kidney transplant recipients were local, a proportion of the deceased donor organs were transported from other jurisdictions. Ethics approval for the conduct of this study was granted by the Sir Charles Gairdner and Osborne Park Health Care Group Human Research Ethics Committee (RGS file number 3027).

### HLA-Typing

High-resolution HLA typing at all HLA loci, including HLA-C, -DRB1, -DQA1, -DQB1, -DPA1 and -DPB1 alleles was performed using ion torrent NGS technology, which uses genomic deoxyribonucleic acid (DNA) to sequence HLA class I (HLA-A, -B, -C) and class II (HLA-DRB1/3/4/5, -DQA1, -DQB1, -DPA1, and -DPB1) genes (ThermoFisher Scientific, Massachusetts, USA). Kidney transplant recipients had high-resolution HLA typing across all HLA loci performed prior to activation on the transplant wait list, whereas for donors, HLA typing was performed using low-intermediate resolution HLA typing on the night of allocation, with updated high-resolution donor HLA typing across extended loci undertaken after transplantation. For donors from Western Australia, HLA typing was performed using the Histospot SSO assay (BagHealth, MC Diagnostics, Germany), and interstate laboratories used LinkSeq rtPCR sequence specific primers (One Lambda, Thermo Fisher Scientific, USA).

### Imputation Using Linkage Disequilibrium

Imputation from the de-identified donor SSO and rtPCR HLA typing was undertaken by an experienced, singular, laboratory scientist without knowledge of the high-resolution NGS typing. Both molecular methods provided one field typing for HLA-DQA1 and DRB3/4/5. We then used linkage equilibrium to facilitate prediction of the most likely 2-field assignment based on local experience and the catalogue of Common and Well Documented (CWD) alleles integrated into the Allele Frequencies Net Database (AFND) (4), Haplostats (5, 6) and the IMGT/HLA Database (7).

### Identification and Calculation of the Number of Eplet Mismatches at HLA-DR and -DQ Alleles

The total number of eplet mismatches at each locus were calculated for NGS- and LD-typing using HLAMatchmaker (Version 2.1 available at www.hlamatchmaker.net). All individual eplet mismatches identified by both NGS-typing and LD-typing were recorded for each donor/recipient pair.

### Identification of Actual Pre-Transplant Donor-Specific Anti-HLA Antibody

Potential DSAs (defined as class I or II DSA with mean fluorescent intensity above 500 on single antigen bead testing using OneLambda LABScreen^®^, reviewed to exclude non-specific background reactivity) were reported for each recipient at time of donor kidney allocation, with the assignment of the DSAs undertaken according to the donor SSO and rtPCR HLA typing. The MFI threshold of 500 to define DSA was chosen as one that reliably predicts worse graft outcomes, having also been validated locally in a previous cohort (8, 9). Cumulative rather than current DSA profiles were used, based on evidence these are more predictive of antibody mediated rejection and consistent with laboratory reporting provided to clinicians at the time of allocation (8, 10). In recipients with pre-existing DSA, the proportions of DSA correctly or incorrectly assigned as actual DSA, confirmed on subsequent high resolution donor HLA typing were reported.

### Statistical Analysis

Baseline characteristics of the study cohort are shown as mean (standard deviation [SD]), number (proportion) or as median (interquartile range [IQR]) where appropriate. NGS-typing was considered the reference standard. Intra-class correlation (expressed as the concordance correlation coefficient with 95% confidence intervals [95% CI]) between NGS-typing and LD-typing was used to examine the consistency and absolute agreement in the number of eplet mismatches. Consistency and absolute agreement were derived from the intraclass correlation coefficients when the systematic differences between measurements for all donor/recipient pairs were considered irrelevant or relevant, respectively. Bland Altman plots were constructed to show the average of the differences and limits of agreement in the total number of eplet mismatches between the NGS typing and LD typing methods.

To assess the absolute differences in the total number of eplet mismatches between the two methods, the proportion of donor/recipient pairs with a difference of 0, 1 to 3, 4 to 6, 7 to 9 and >10 eplet mismatches at HLA-DR and -DQ alleles was determined. Fisher’s exact test was used to compare categories. Analyses were undertaken using SAS 9.4 (SAS Institute, Cary, NCNC) and R 3.6 (R Core Team, Vienna, Austria).

## Results

### Baseline Characteristics

There were 94 kidney transplant recipients who received kidneys from 77 deceased donors, of which 55 (71%) and 22 (29%) were local and interstate donors, respectively. Of the 94 donor/recipient pairs, 73 (78%) recipients and 56 (of 78 donors, 72%) donors were of Caucasian ethnicity by self-report (**Table 1**). The median [IQR] age of the recipients was 55 [46, 61] years, and the median age and Kidney Donor Profile Index (KDPI) of the donors were 47 [38, 57] and 44 [25, 66], respectively. The median [IQR] number of class I and class II eplet mismatches were 16 [11, 19] and 38 [26, 48], respectively.

**Table 1:** Baseline characteristics of the study cohort.

Recipient (n = 94)	
Age	55 [46, 61]
Male	62 (66%)
Ethnicity	
Caucasian	73 (78%)
Asian	13 (14%)
Aboriginal and Torres Strait Islander	5 (5%)
Other	3 (3%)
**Donor (n = 78)**	
Age	47 [38, 57]
Male	43 (55%)
KDPI	44 [25, 66]
**Ethnicity**	
Caucasian	56 (72%)
Asian	11 (14%)
Aboriginal and Torres Strait Islander	5 (6%)
Other	6 (8%)

Results expressed as median [inter-quartile range] or number (percentage).

KDPI, kidney donor profile index.

### Concordance and Agreement

The concordance coefficient for total class I eplet mismatches between NGS-typing and LD-typing was 0.994 (95%CI 0.992 to 0.996), for total class II mismatches the concordance coefficient was 0.991 (95% 0.986 to 0.993).

**Figure 1:**
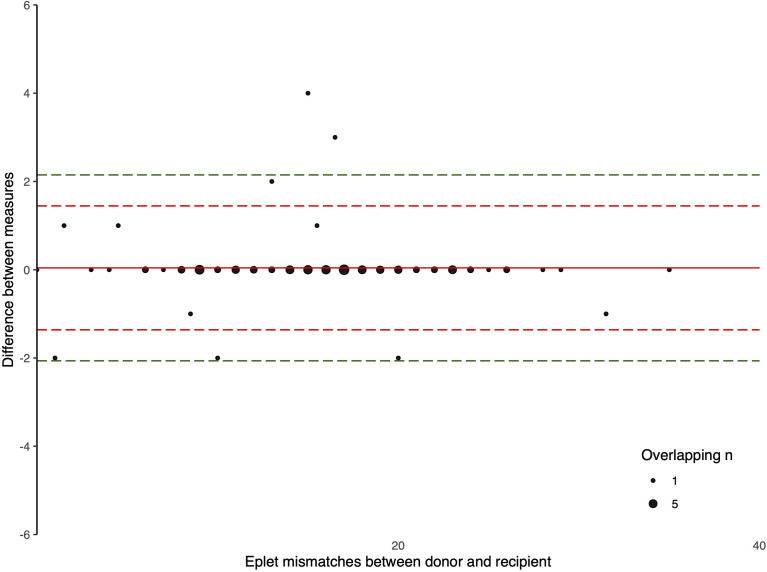
Bland Altman Plot – total HLA class I eplet mismatches. A positive result indicates a greater number of eplet mismatches identified by NGS-typing compared to LD-typing. The red and green dashed lines represent 2 and 3 standard deviations from mean respectively.

**Figure 2:**
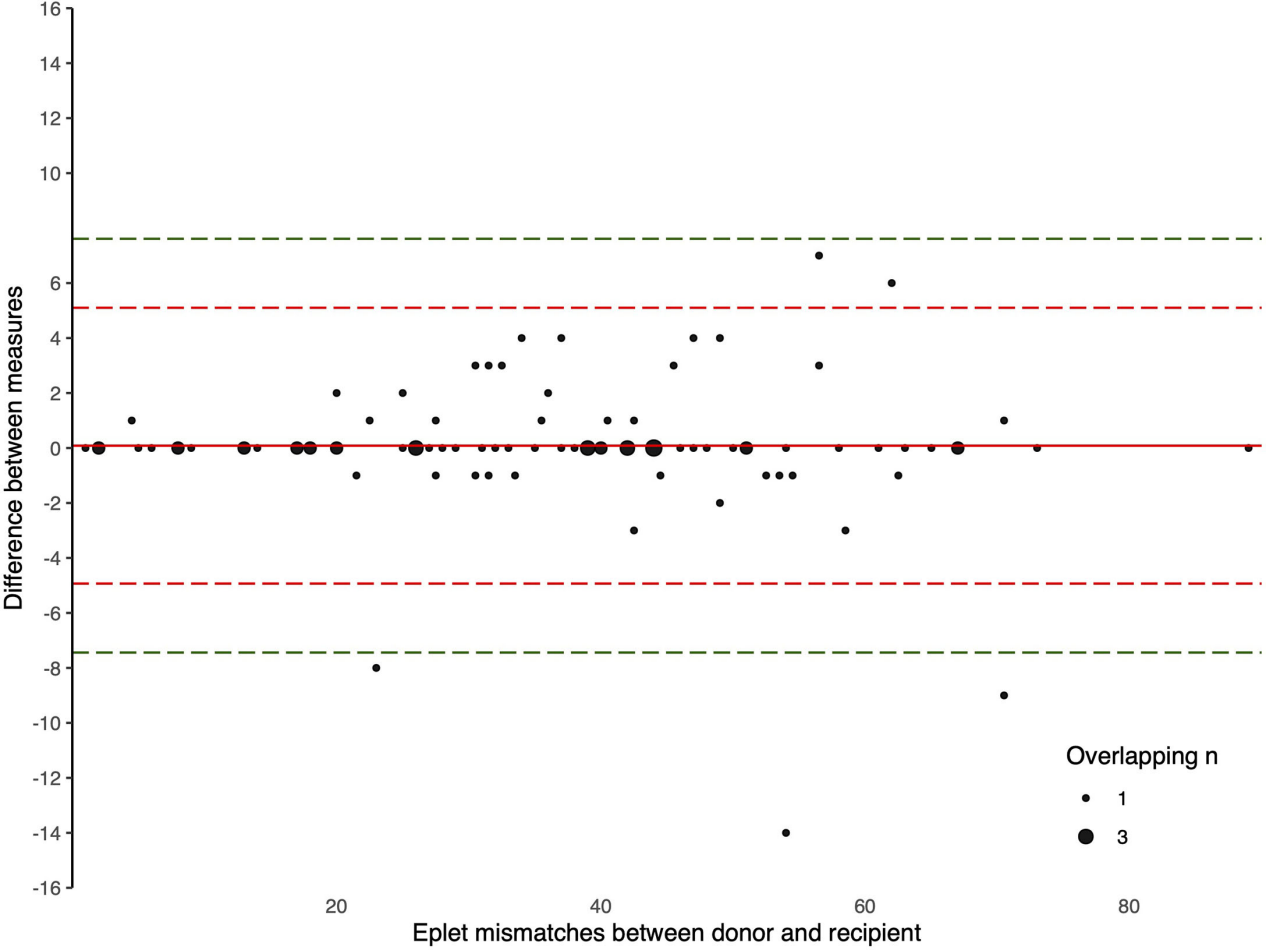
Bland Altman Plot – total HLA class II eplet mismatches. A positive result indicates a greater number of eplet mismatches identified by NGS-typing compared to LD-typing. The red and green dashed lines represent 2 and 3 standard deviations from mean respectively.

The Bland-Altman plots comparing the number of eplet mismatches calculated using NGS-typing versus LD-typing for class I and II mismatches, in **Figures 1**, **2**, respectively. The mean difference (bias) between NGS-typing and LD-typing methods for class I mismatches was 0.0 (95%CI -0.1 to 0.2), the 95% limits of agreement were -1.3 (95%CI -1.6 to -1.1) to 1.4 (95%CI 1.2 to 1.7). The mean difference for class II mismatches was 0.1 (95%CI -0.4 to 0.6). The 95% limits of agreement for class II mismatches from -4.8 (95%CI -5.7 to -3.9) to 5.0 (95%CI 4.1 to 5.9).

The 95% limits of agreement for Caucasian donors were -1.3 (95%CI -1.6 to -1.0) to 1.4 (95%CI 1.2 to 1.7) for class I mismatches, and -3.2 (95%CI -3.8 to -2.5) to 3.5 (95%CI 2.8 to 4.1) for class II mismatches; compared to 95% limits of agreement for non-Caucasian donors of -1.5 (95%CI -2.3 to -0.8) to 1.5 (95%CI 0.8 to 2.3) for class I mismatches, and -3.3 (-5.2 to -1.3) to 5.5 (95%CI 3.5 to 7.5) for class II mismatches. The 95% limits of agreement for those donors with SSO and rtPCR HLA typing were similar (**Supplementary Material, Tables S1–S2, Figures S1–4**).

### Absolute Difference in the Number of Eplet Mismatches

There were 44 (47%) donor/recipient pairs with differences in the number of eplet mismatches identified by NGS-typing and LD-typing. The greatest discrepancy in the number of class I eplet mismatches for any patient was 4, and for class II was 14, with 4 patients having a difference of 7 or more (**Table 2**). Allelic differences between NGS and LD methods resulting in differences in eplet matching occurred in 5 donors (**Supplementary Material, Table S3**). For these donors, when the HLA typing was repeated in the recipient Western Australian laboratory different results, consistent with the NGS-typing, were obtained for 4 donors. Removing these donors from the dataset made no material difference to the results (**Supplementary Material, Table S4**).

**Table 2:** Absolute agreement in the number of eplet mismatches using NGS versus LD-typing.

Number of mismatches	Class I	Class II
0	83 (88%)	57 (61%)
1 to 3	10 (11%)	28 (30%)
4 to 6	1 (1%)	5 (5%)
7 to 9	–	3 (3%)
10 to 15	–	1 (1%)

Results are presented as n (%) from pairs in total (N).

The frequency of any difference between NGS-typing and LD-typing was higher among non-Caucasian compared to Caucasian donors; however, the difference was not statistically significant (24% vs 10% for class I [p=0.1] and 53% vs 34% for class II [p=0.2], among non-Caucasian and Caucasian donors, respectively). Three donors (transplanted into four recipients) and two recipients had HLA alleles resolved by NGS-typing that were not found in HLA Matchmaker (**Supplementary Material, Table S5**). Alleles with between 1- and 6-base pair discrepancies were imputed. Two of these donors were Aboriginal and Torres Strait Islander Peoples, and one donor was from South-East Asia. Of the recipients with alleles not found in HLA Matchmaker, one was an Aboriginal and Torres Strait Islander person and the other was Caucasian.

### Identification of “Discordant” HLA-DR and -DQ Eplet Mismatches

For 15 donor/recipient pairs, additional discrepancies in the exact eplet mismatches were identified, beyond differences in the simple sum of the calculated number of eplet mismatches by NGS-typing and LD-typing (**Supplementary Material, Table S6**). For the remaining 22 pairs with discordant results in the number of eplet mismatches identified by NGS-typing compared to LD-typing, the simple sum of eplet mismatches was sufficient to identify the maximal discordance between the two methods. Haplotype frequencies for recipient and donor of one pair, two recipients of Asian background, and one other donor of did not have well documented DQA1 associations with their corresponding DQB1.

### Assignment of Donor-Specific Anti-HLA Antibody

Of the study cohort, 34 (36%) recipients had detectable potential pre-transplant DSAs with MFI above 500 at time of donor kidney allocation, with 26 (28%) recipients having multiple potential DSAs. Most recipients had correct assignment of all/some actual DSAs (n=26, 54% of recipients with DSA), but indeterminate assignment of potential DSAs were observed among 8 recipients when there would otherwise have been no actual DSA detected (24% of recipients with DSA) (11 of 13 [85%] class II DSAs). Assignments of class I and II pre-transplant DSAs, with the corresponding SSO and rtPCR HLA typing are detailed in **Table 3**. These were more likely to occur in recipients with multiple allele-specific DSAs, which was more apparent for DSA directed against class II alleles (HLA-DRB1, -DQA1, -DQB1). All the 48 potential DSA subsequently re-classified following NGS-typing, were anti-HLA antibodies identified with low-intermediate resolution donor typing but directed to a different allele. The median MFI of indeterminate DSA was 1411 [1053, 2337]. Among the 26 recipients with indeterminate DSA, 14 (54%) of these had at least one incorrect DSA to HLA-A/-B/-DRB1 loci. If the presence of any pre-transplant DSA with MFI above 2000 was considered a contraindication for donor kidney allocation, 4 (4%) recipients would have been ineligible to receive the donor kidney, with DSAs to HLA-DRB1, -DQA1 and -DQB1 incorrectly assigned.

**Table 3:** The assignment of actual pre-transplant donor-specific anti-human leukocyte antigen antibodies according to intermediate resolution donor typing at time of donor kidney allocation.

Patients	Potential donor-specific anti-HLA antibody (MFI)	Intermediate-typing	Method	NGS-typing
**1**	DQA1*05:03 (2085); DQA1*05:05 (2388) DQB1*03:01 (2480)	DQA1*05 DQB1*03	rtPCR	DQA1*05:03 DQB1*03:01
**2**	DRB5*01:01 (2348)	DRB5*01	rtPCR	DRB5*01:01
**3**	DRB1*04:01 (1116); DRB1*04:03 (1008); DRB1*04:04 (2441) DQA1*03:01 (2012); DQA1*03:02 (5242) DQB1*03:02 (2399) DPB1*06:01, DPB1*04:02 (1181)	DRB1*04 DQA1*03 DQB1*03 DPB1*06:01; DPB1*04:02	rtPCR	DRB1*04:04 DQA1*03:01 DQB1*03:01 DPB1*06:01 DPB1*04:02
**4**	DRB1*04:04 (1594)	DRB1*04	rtPCR	DRB1*04:01 DRB1*04:08
**5**	DRB1*07:01 (1334)	DRB1*07	SSO	DRB1*07:01
**6**	DRB4*01:01 (2604); DRB4*01:03 (2477)	DRB4*01	rtPCR	DRB4*01:03
**7**	DQB1*05:01 (1231)	DQB1*05	SSO	DQB1*05:01
**8**	DRB1*14:01 (1286); DRB1*14:54 (1011)	DRB1*14	SSO	DRB1*14:03
**9**	DQA1*05:03 (2026); DQA1*05:05 (1899)	DQA1*05	SSO	DQA1*05:01
**10**	DRB1*04:03 (1016)	DRB1*04	SSO	DRB1*04:03
**11**	DRB1*04:04 (807)	DRB1*04	SSO	DRB1*04:01
**12**	DQA1*05:01 (2242) DPB1*11:01 (562)	DQA1*05 DPB1*11	SSO	DQA1*05:01 DPB1*11:01
**13**	C*15:02 (539) DRB1*15:01 (903)	C*15 DRB1*15	SSO	C*15:02 DRB1*15:01
**14**	DRB1*04:04 (1621) DRB5*01:01 (732)	DRB1*04 DRB5*01	SSO	DRB1*04:01 DRB5*01:01
**15**	DRB4*01:03 (506) DQB1*03:02 (754)	DRB4*01 DQB1*03:02	SSO	DRB4*01:03 DQB1*03:02
**16**	DRB1*16:01 (1837); DRB1*16:02 (1736) DQA1*01:01 (5551); DQA1*01:02 (8195); DQA1*01:03 (5410) DQB1*03:01 (626); DQB1*05:01 (5551): DQB1*05:02 (2754)	DRB1*16 DQA1:01 DQB1*03; DQB1*05	rtPCR	DRB1*16:02 DQA1*01:02 DQB1*03:01 DQB1*05:02
**17**	A*02:01 (820); A*02:06 (862) B*13:01 (854); B*13:02 (657)	A*02 B*13	SSO	A*02:01 B*13:02
**18**	DRB3*03:01 (926) DQB1*06:01 (834)	DRB3*03 DQB1*06	SSO	DRB3*03:01 DQB1*06:04
**19**	C*04:01 (1214)	C*04	SSO	C*04:01
**20**	DRB1*04:04 (3682) DQA1*03:03 (18654) DQB1*02:01 (16204)	DRB1*04 DQA1*03 DQB1*02	SSO	DRB1*04:01 DQA1*03:01 DQB1*02:02
**21**	B*07:02 (17570) DQB1*03:02 (2106); DQB1*03:03 (2333)	B*07 DQB1*03:03	SSO	B*07:02 DQB1*03:03
**22**	C*07:02 (1720) DQA1*02:01 (1333) DQB1*02:01 (830); DQB1*02:02 (1065)	C*07 DQA1*02 DQB1*02:01; DQB1*02:02	SSO	C*07:01 DQA1*02:01 DQB1*02:01 DQB1*02:02
**23**	DRB4*01:01 (1610); DRB4*01:03 (1215)	DRB4*01	SSO	DRB4*01:01 DRB4*01:03
**24**	A*68:02 (1107) DQA1*05:01 (2409)	A*68 DQA1*05	SSO	A*68:01 DQA1*05:05
**25**	B*27:05 (2511); B*27:08 (1220) C*07:02 (2297) DRB1*14:01 (1420); DRB1*14:54 (1207) DRB3*03:01 (23471); DRB3*02:02 (19698) DPA1*01:04	B*27 C*07 DRB1*14 DRB3*03:02 DPA1*01	SSO	B*27:05 C*07:02 DRB1*14:54 DRB3*02:02 DPA1*01:03
**26**	C*02:02 (1950); C*06:02 (1479)	C*02; C*06	SSO	C*02:02 C*06:02
**27**	B*18:01 (1208) DQA1*03:01 (20173); DQA1*03:02 (9186); DQA1:03:03 (5319)	B*18 DQA1*03	rtPCR	B*18:01 DQA1*03:02
**28**	C*07:02 (2063) DRB1*04:01 (1131); DRB1*04:02 (2570); DRB1*04:03 (1169); DRB1*04:04 (1832); DRB1*04:05 (1533); DRB1*11:01 (2009) DQA1*05:01 (5097)	C*07 DRB1*04; DRB1*11 DQA1*05	SSO	C*07:02 DRB1*04:01 DRB1*11:01 DQA*05:05
**29**	B*57:01 (1343); B*57:03 (1650) DQA1*05:01 (2539)	B*57 DQA1*05	SSO	B*57:01 DQA1*05:01
**30**	C*12:03 (1047)	C*12	SSO	C*12:02
**31**	DRB1*13:03 (2350)	DRB1*13	SSO	DRB1*13:01
**32**	DRB1*04:01 (1099); DRB1*04:03 (1121)	DRB1*04	SSO	DRB1*04:03
**33**	A*02:01 (2732); A*02:03 (1890); A*02:06 (2257)	A*02	SSO	A*02:01
**34**	B*52:01 (2034) DRB5*01:01 (3441) DQA1*05:03 (9536); DQA1*05:05 (9558) DQB1*06:01 (1484); DQB1*06:09 (1581)	B*52 DRB5*01 DQA1*05 DQB1*06	rtPCR	B*52:01 DRB5*01:02 DQA1*05:01 DQB1*06:01

Table showing the kidney transplant recipients with reported donor-specific anti-HLA antibody at time of transplantation, with corresponding intermediate resolution allele-specific typing available at time of donor kidney allocation. Actual donor-specific anti-HLA antibodies (bolded and underlined) are confirmed by high resolution donor HLA typing.

Human leukocyte antigen (HLA); mean fluorescent intensity (MFI); real-time polymerase chain reaction (rtPCR); sequence-specific oligonucleotide (SSO).

## Discussion

This contemporary study highlights the clinical impact of limitations in donor HLA typing at time of organ allocation obtained by SSO and rtPCR typing. The incorrect number of eplet mismatches was assigned to nearly 50% of recipients, and 24% of potential DSA would have been incorrectly assigned. The impact of the former discrepancy will be offset by narrow limits of agreement between the two methods. However, the incorrect assignment of potential DSAs poses significant challenges for clinicians, as the decision for accepting or declining potential kidney offers may be influenced by the specificity of even a single antibody, depending upon its strength and the recipient’s clinical and immunologic profile.

Eplet matching as a means of improving donor-recipient HLA matching has already been successfully implemented by several transplant programs internationally (11, 12). While more sophisticated methods of applying eplet matching or epitope matching will evolve, current programs rely on thresholds of eplet mismatches (13). Given the 95% limits of agreement of -1.3 to 1.4 for class I and -4.8 to 5.0 for class II, relatively few recipients will move across a threshold; although for recipients with a narrow potential donor profile, even the decline of one appropriate offer may be clinically significant.

Our finding that a high proportion of DSA remain indeterminate using low-intermediate resolution donor HLA typing alone is a concern, as the pool of highly sensitized recipients with complex anti-HLA antibody profiles grows. Indeterminate results were more common than incorrectly assigned DSA; however, given this distinction is not known at allocation both are clinically important and potentially impact the allocation or acceptance of donor kidneys. This result is consistent with other studies that have shown not only are DSA commonly misclassified, but that long-term graft outcomes among donors with indeterminate DSA identified on LD-typing but to a different allele on NGS-typing are similar to those without any indication of DSA (14). The study cohort did not examine refused organ offers and it is possible that the impact of indeterminate DSA may be greater again among the total potential donor pool.

The definition and weighting of common allelic associations, inherent to LD-typing, favors individuals from common racial groups. Beyond this, for some minority groups there are a lack of HLA frequency data and likely some alleles either not yet fully defined or incorporated into standard laboratory platforms, including solid phase assays to detect DSA. This problems is exacerbated at loci such as HLA-DQA1 and -DRB3/4/5 less commonly typed in registry data used to derive haplotype frequencies and linkages (6). In this study, three donors contributing four kidneys, and two recipients had alleles not present in the current version of HLA Matchmaker. Three of these were Aboriginal and Torres Strait Islander Peoples and one was from South-East Asia. These findings are consistent with those from Scientific Registry of Transplant Recipients (SRTR) whereby the inclusion of ethnicity has been shown to improve the accuracy of HLA imputation (15). In this study differences between LD-typing and NGS-typing were more common among non-Caucasian donors. The result was not statistically significant, but this may reflect a lack of statistical power rather than the absence of a true relationship.

This study includes a consecutive series of deceased donor kidney transplants from a single-center with both low-intermediate resolution HLA typing performed at the time of transplantation, and retrospective NGS-typing following transplantation. Hence, the results accurately reflect the impact of current standard of care HLA typing methods used in the allocation of deceased donor organs. The assignment of alleles by linkage disequilibrium were undertaken by a senior scientist, also consistent with current clinical practice. This may lead to a smaller error in LD-typing compared to studies using the fully automated imputation method contained within HLA Matchmaker to assign high resolution typing. For a small number of donors the SSO and rtPCR HLA typing was discrepant with the NGS-typing, rather than lacking resolution. While subsequent results indicated an error in the HLA typing at the time of allocation, we elected to leave these in the dataset to reflect the error rate observed in routine clinical practice. Limitations inherent to this type of study include subsequent advances in the methods used, including the ability of SSO and rtPCR HLA typing to resolve an expanding number of alleles and updates to HLA Matchmaker.

There are potential solutions in development for the problems identified in this study. Perhaps the most direct are rapid NGS typing methods that can be completed in 4.5 hours with equivalent accuracy across 11-loci (16). However, these are not yet widely available in clinical practice and access to this method, including the required operator expertise, is unlikely to be available in resource constrained settings soon. With regards to eplet matching, threshold-based allocation methods are likely to be superseded as data about the relationship between eplet load and risk expand. This includes the identification of high-risk eplet mismatches acknowledging that not all mismatches are equally immunogenic (13). Beyond this, the differential impact of DSA to different HLA loci and eplets on graft function and the development of transplant glomerulopathy needs to be better understood, especially at low expression loci such as DRB3/4/5 (17, 18). Raw MFI values are at best semi-quantitative. There is large inter-assay and inter-laboratory variation, moreover antigen density varies across loci on single antigen bead testing, as does the expression of antigens at different loci and clinical impact of DSA *in-vivo* (19, 20). Nevertheless, we suggest that rapid NGS methods be further explored for application in deceased donor allocation to overcome the problems with LD-typing highlighted in this study.

In summary, the use of linkage disequilibrium to impute 2-field HLA allele type across extended loci has limitations both in accurately identifying eplet mismatches and assigning DSA. Further research is required to better understand and mitigate these limitations arising from SSO and rtPCR HLA typing, until rapid NGS methods are routinely used for solid organ donor allocation.

## Data Availability Statement

The original contributions presented in the study are included in the article/**Supplementary Material**, further inquiries can be directed to the corresponding authors.

## Ethics Statement

The studies involving human participants were reviewed and approved by Sir Charles Gairdner and Osborne Park Health Care Group Human Research Ethics Committee. Written informed consent for participation was not required for this study in accordance with the national legislation and the institutional requirements.

## Author Contributions

All authors contributed to the study design, data interpretation, and manuscript preparation. AT performed the LD-typing and eplet analysis. NL performed the statistical analysis. All authors approved the final version for publication and agree to be accountable for all aspects of the work.

## Funding

NL work on this study was supported by a Clinician Research Fellowship from the Department of Health and Raine Medical Research Foundation. GW is a recipient of the NHMRC Career Development Fellowship APP 1447657 and NHMRC Leadership Fellow APP 1195414.

## Conflict of Interest

The authors declare that the research was conducted in the absence of any commercial or financial relationships that could be construed as a potential conflict of interest.

## Publisher’s Note

All claims expressed in this article are solely those of the authors and do not necessarily represent those of their affiliated organizations, or those of the publisher, the editors and the reviewers. Any product that may be evaluated in this article, or claim that may be made by its manufacturer, is not guaranteed or endorsed by the publisher.
