# Supplementary material for: The Accuracy of Sequence-Specific Oligonucleotide and Real-Time Polymerase Chain Reaction HLA Typing in Determining the Presence of Pre-Transplant Donor-Specific Anti-HLA Antibodies and Total Eplet Mismatches for Deceased Donor Kidney Transplantation
Source: Front Immunol. 2022 Jun 20;13:844438. doi: 10.3389/fimmu.2022.844438 (PMC9253866; doi:10.3389/fimmu.2022.844438)
Supplement: Supplementary file 1 [file DataSheet_1.pdf]

Table S1 Mean class I difference and limits of agreement by donor typing method

	**Complete**	**SSO**	**rtPCR**
Lower limit of agreement (95% CI)	-1.3 (-1.6 to -1.1)	-0.9 (-1.0 to -0.7)	-2.0 (-2.9 to -1.1)
Mean difference (95% CI)	0.0 (-0.1 to 0.2)	-0.1 (-0.2 to 0.0)	0.3 (-0.2 to 0.9)
Upper limit of agreement (95% CI)	1.4 (1.2 to 1.7)	0.7 (0.6 to 0.9)	2.7 (1.8 to 3.6)

Real-time polymerase chain reaction (rtPCR); sequence-specific oligonucleotide (SSO).

Table S2 Mean class II difference and limits of agreement by donor typing method

	**Complete**	**SSO**	**rtPCR**
Lower limit of agreement (95% CI)	-4.8 (-5.7 to -3.9)	-5.1 (-6.2 to -4.0)	-4.1 (-5.7 to -2.4)
Mean difference (95% CI)	0.1 (-0.4 to 0.6)	0.0 (-0.6 to 0.6)	0.3 (-0.7 to 1.2)
Upper limit of agreement (95% CI)	5.0 (4.1 to 5.9)	5.1 (4.1 to 6.2)	4.6 (2.9 to 6.2)

Real-time polymerase chain reaction (rtPCR); sequence-specific oligonucleotide (SSO).

Table S3 Donors with discordant HLA-typing by SSO and NGS

**SSO-typing**	**NGS-typing**
DPB1*45:01,*46:01	DPB1*02:01,*03:01
DPA1*01	DPA1*01:03,*02:01
DPB1*14,*17	DPB1*03:01,*17:01
DPA1*02,*04	DPA1*02:02
DRB3*01,*02	DRB3*02:02

SSO – sequence-specific oligonucleotide; NGS – Next Generation Sequencing.

Table S4 Mean difference and limits of agreement with discordant typing removed

	**Class I**	**Class II**
Lower limit of agreement (95% CI)	-1.1 (-1.4 to -0.9)	-4.9 (-5.8 to -4.0)
Mean difference (95% CI)	0.0 (-0.1 to 0.1)	0.0 (-0.5 to 0.5)
Upper limit of agreement (95% CI)	1.1 (0.9 to 1.4)	4.9 (4.0 to 5.8)

Five donor/recipient pairs in Table S3 removed.

Table S5 HLA alleles resolved by NGS not present in HLAMatchmaker

**Donors**
DRB1*14:09 does not correspond with any G group, sequence alignment performed with DRB1*14:01, DRB1*14:03, DRB1*14:04, DRB1*14:08. Closest alignment with DRB1*14:02 - 1 bp difference
A*74:05 does not correspond with any G group, sequence alignment performed with A*74:01, A*74:02, A*74:03, A*74:06 and A*74:08. Closest alignment with A*74:01 - 1 bp difference<br>DRB1*11:129= DRB1*11:06 does not correspond with any G group, sequence alignment performed with DRB1*011 Closest alignment with DRB1*11:06 -1 bp difference
DRB1*03:124 does not correspond with any G group, sequence alignment performed with DRB1*03 Closest alignment with DRB1*03:01 -1 bp difference
**Recipients**
DPB1*112:01 does not correspond with any G group, sequence alignment performed with DPB1*02:01, DPB1*02:02, DPB1*03:01, DPB1*04:01, DPB1*04:02, DPB1*15:01. Closest alignment with DPB1*04:01 -2 bp difference
DRB1*04:12 does not correspond with any G group, sequence alignment performed with DRB1*04:01, DRB1*04:02, DRB1*04:03, DRB1*04:04, DRB1*04:05, DRB1*04:06, DRB1*04:07, DRB1*04:08, DRB1*04:09, DRB1*04:10, DRB1*04:11, DRB1*04:13 Closest alignment with DRB1*04:10 -6 bp difference

Table S6 Comparing the actual HLA-DR and HLA-DQ mismatched eplets identified by Next Generation Sequencing (NGS) typing and linkage disequilibrium. Examples of donors-recipient pairs where there is a disagreement (difference in predicted eplet mismatches of ≥1) between the two methods.

	NGS	PREDICTED	DIFFERENCE IN NUMBER HLA-DR+DQ EPLET MM	ACTUAL NUMBER HLA-DR+DQ EPLET MM	HLA-DR EPLET MM	HLA-DQ EPLET MM	DONOR HLA TYPING METHOD
**PAIR 1**	21	21	0	2	16Y*, 57S, 67F, 74L	26G*, 45EV*, 52PR*, 55RL*, 74SV*, 70ED*, 9F, 14AM, 26Y, 26G, 56PD, 70ED, 66IT, 75IL, **160AD**, **160D**	SSO
**PAIR 2#**	45	43	2	10	11STS*, 74R*, 77N*, 96HK*, 98Q*, 104A*, 67LQ*, 13SE, **28E**, **30H**, 32H, **37NV**, **37YA**, 71K, 73G, 85VV, 96H, 140TV, 149H, **189S**, **37FL2**, **57V**, **60S**, **183A**	45GE*, 55LPA*, 74AVR*, 125A*, 40GR*, 47CL*, 53QF*, 75S*, 163E*, 175K*, 9F, 13GM, 26L, 37YA, 56PA, 70GT, 18S, 45VL, 61FT, 66IL, 76L, 80S, **129H**, 160AE	SSO
**PAIR 3#**	29	28	1	3	70QT*, 96EV*, 96ES*, 30C, 37SV, **37FL2**, 74A, **183A**, **28E**	26G*, 52PQ*, 56PV*, 52PR*, 74SR*, 74SV*, 85VG*, 116I*, 125S*, 14GL, 26G, 67VG, 85VY, 18F, 41RA, 52SK, 64RM, 80Y, 130S, 160AD	SSO
**PAIR 4#**	39	39	0	6	96EV*, 108T*, 142M*, 347F*, 85VG*, 96EN*, 104ART*, 28H, 37DL, 267I, 67F, 70Q, 71A, 74A, 120S, 133RS, 180V	52PQ*, 52PR*, 85VG*, 140A*, 182S*, 9F, 56PD, 67VG, 70GT, 125GQ, 185T, 18F, **25FT**, **41KA**, 52SK, 64RM, **130A**, 80Y, 187A, **25YT**, **41RA**, **130S**	rtPCR
**PAIR 5#**	40	40	0	6	4Q*, 11STS*, 18L*, 25Q*, 26WN*, 48Q*, 40YNL*,	45GE*, 55LPA*, 74AVR*, 47KHL*, 40ERV*, 13GM,	SSO

					74R*, 81Y*, 12KYK, 28D, **30Y**, 31I, 32Y, 37NV, **37FV**, 37FL, 267I, 71E, 74E, **85VV**, 98E, 183A, **133RS**, **140AV**, **189S**	26L, 56PA, 66D, 66DR, 70GT, 125GQ, 135G, 41KA, 66IL, 130A	
**PAIR 6#**	35	38	3	7	96EV*, 108T*, 142M*, 396EN*, 104ART*, 28D, 37SV, **37DL2**, **133RS**, **6C**, **67I**, **70Q**, **71A**, **157I**	26G*, 52PQ*, 52PR*, 74SR*, 74SV*, 77R*, 85VG*, 140A*, 116I*, 125S*, 182S*, 14GL, 26G, 56PS, 67VG, 75V, 85VY, 125SH, 18F, 25YT, 41RA, 52SK, 64RM, 80Y, 130S	rtPCR
**PAIR 7**	8	7	1	3	37FV, **57V**, **60S**, **28E**	56PV*, 70RT, 85VY, 86G, 130Q	

*#denotes recipients of Asian ethnicity, *denotes mismatched eplet with high risk of immunisation defined by both SSO/rtPCR and NGS, **red bold colour** denotes mismatched eplet identified by NGS method only and **bold blue colour** denotes mismatched eplet identified by linkage disequilibrium method only*

Figure S1 Bland Altman Plot – total HLA class I eplet mismatches – SSO donor HLA typing

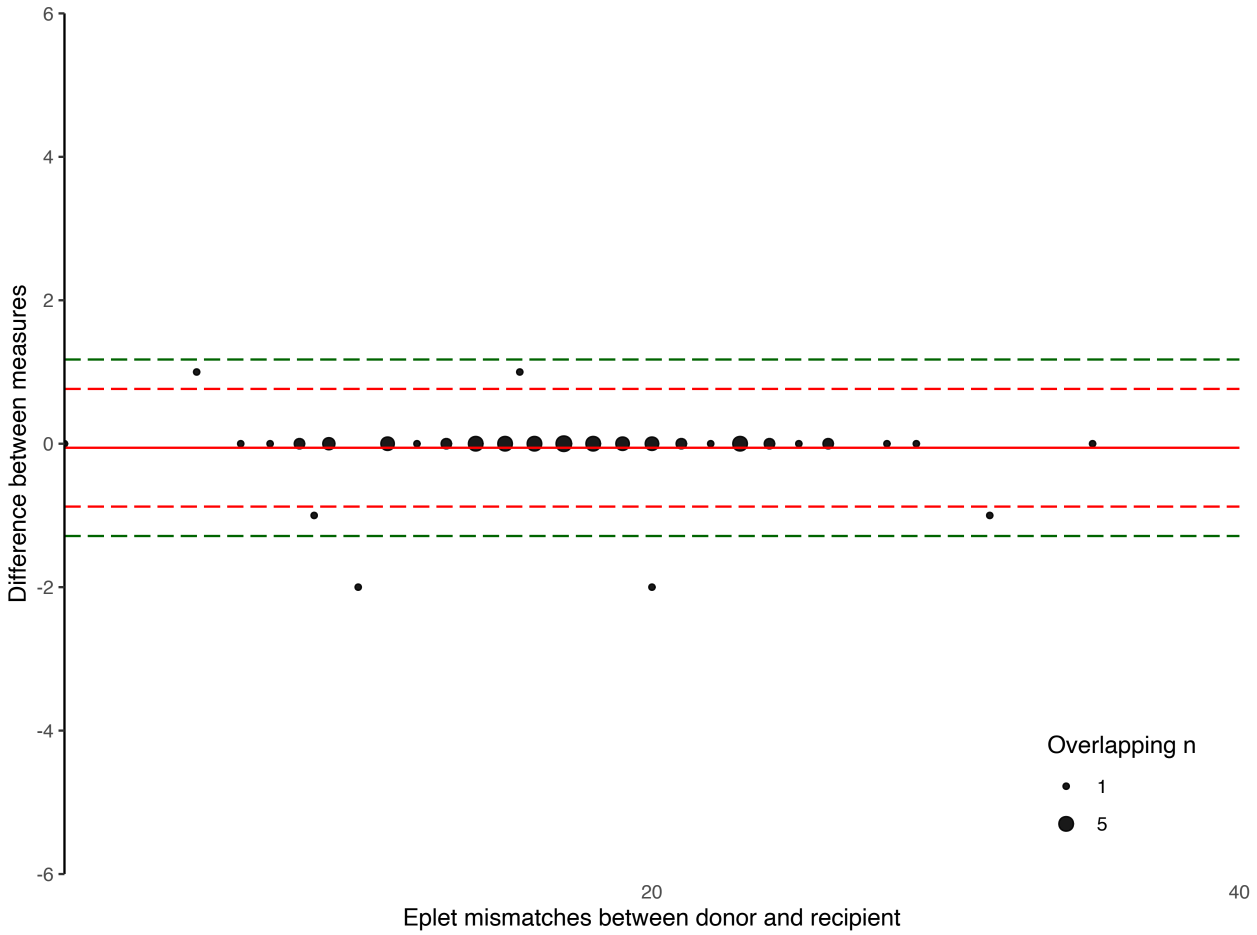

A positive result indicates a greater number of eplet mismatches identified by NGS-typing compared to LD-typing. The red and green dashed lines represent 2 and 3 standard deviations from mean respectively.

Figure S2 Bland Altman Plot – total HLA class II eplet mismatches – SSO donor HLA typing

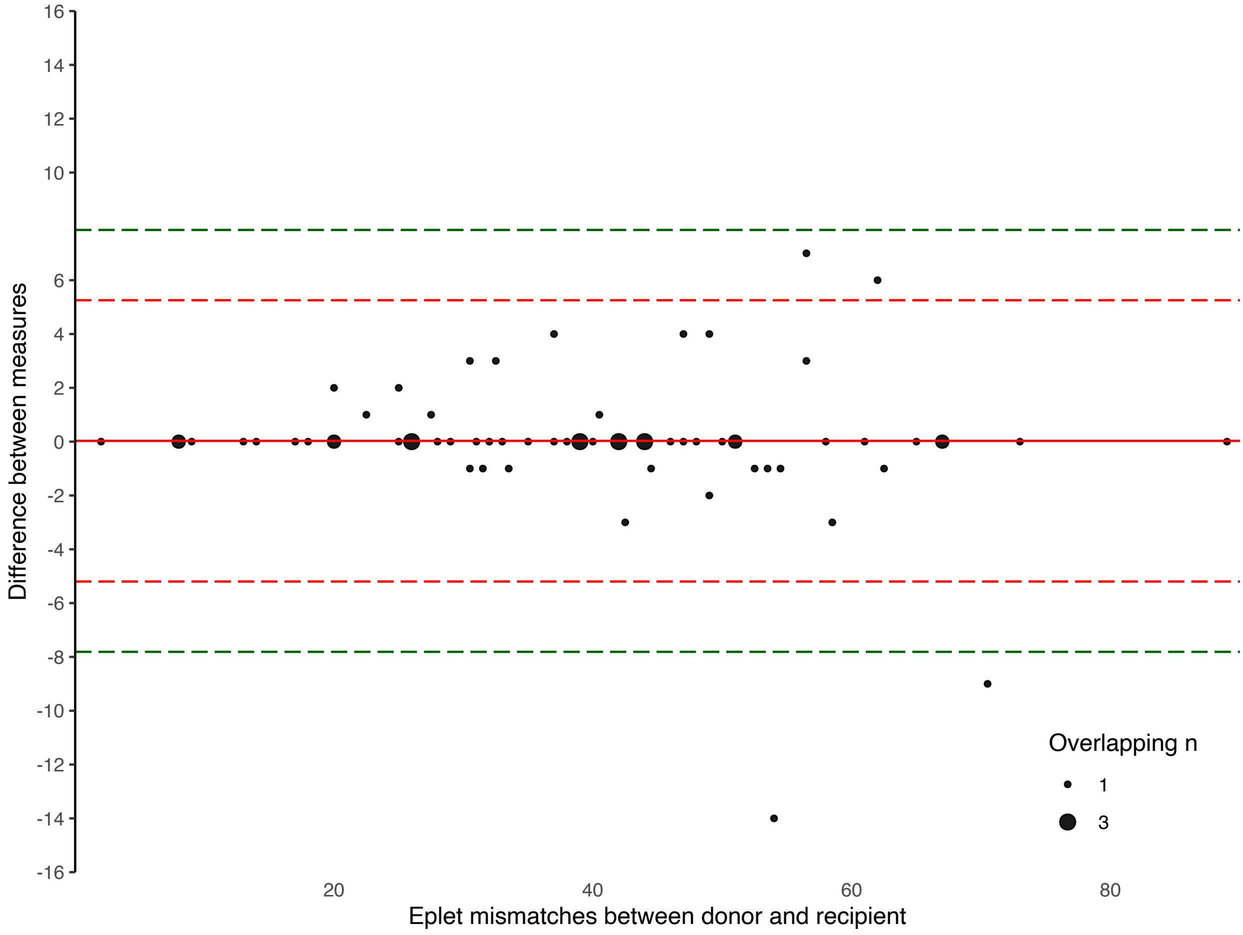

A positive result indicates a greater number of eplet mismatches identified by NGS-typing compared to LD-typing. The red and green dashed lines represent 2 and 3 standard deviations from mean respectively.

Figure S3 Bland Altman Plot – total HLA class I eplet mismatches – rtPCR donor HLA typing

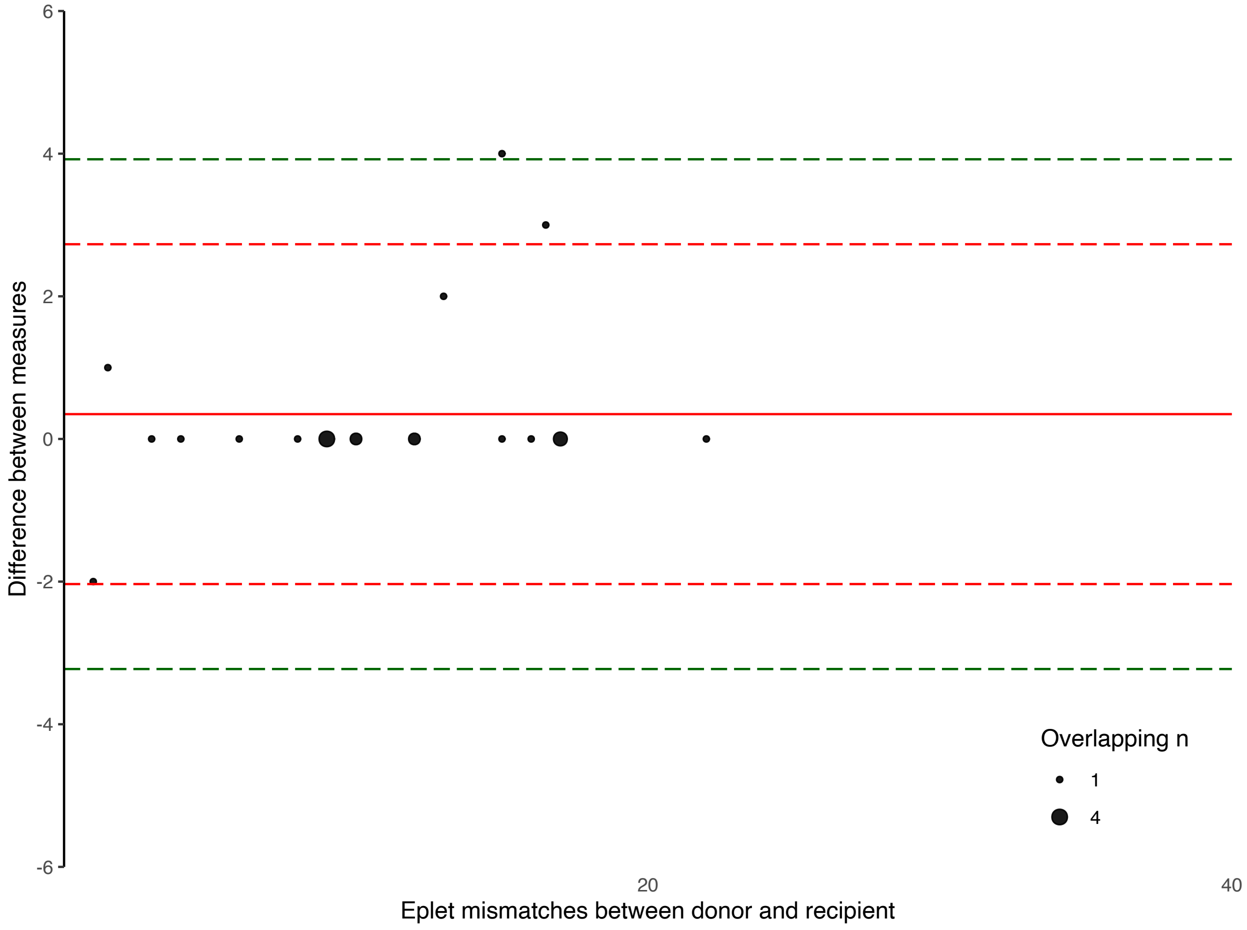

A positive result indicates a greater number of eplet mismatches identified by NGS-typing compared to LD-typing. The red and green dashed lines represent 2 and 3 standard deviations from mean respectively.

Figure S4 Bland Altman Plot – total HLA class II eplet mismatches – rtPCR donor HLA typing

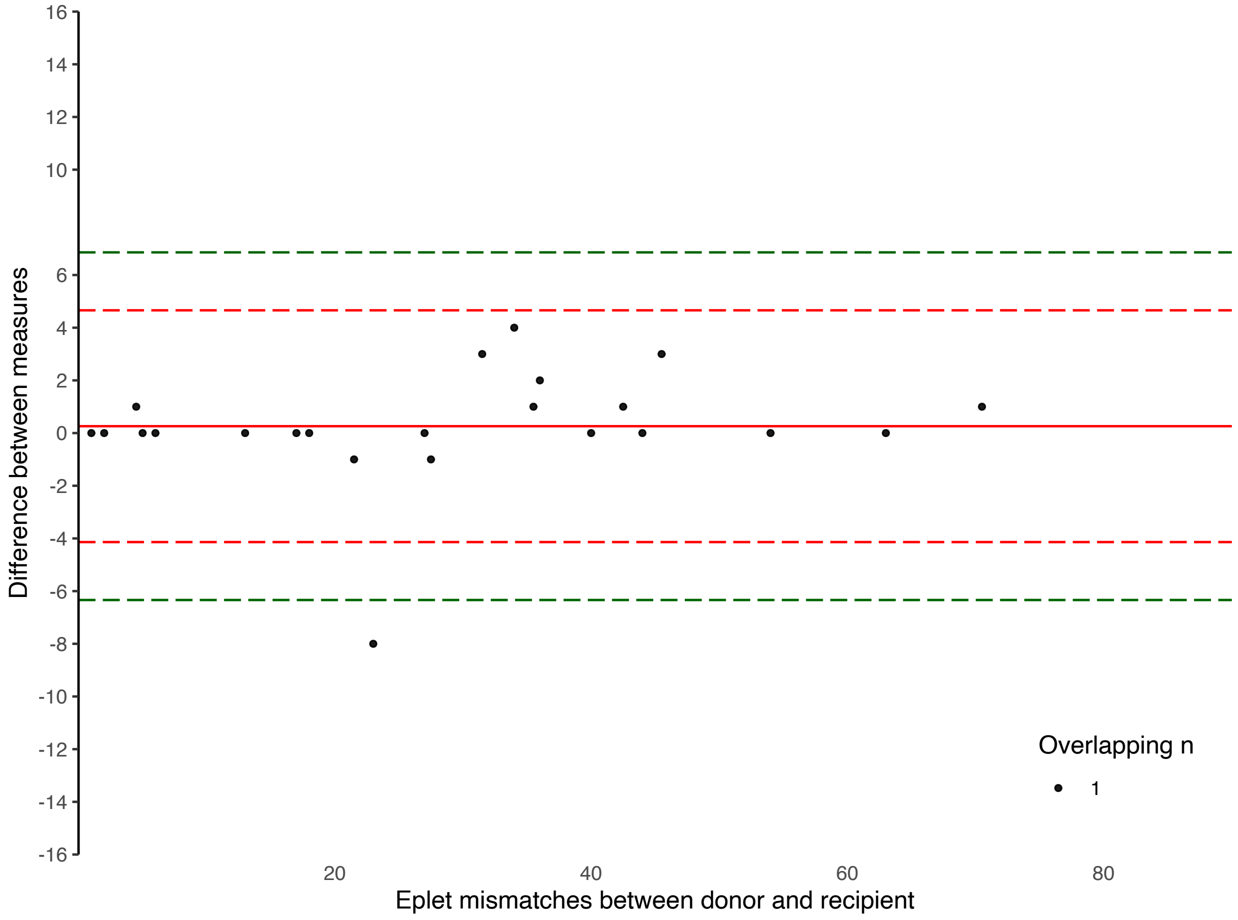

A positive result indicates a greater number of eplet mismatches identified by NGS-typing compared to LD-typing. The red and green dashed lines represent 2 and 3 standard deviations from mean respectively.
